# CsDETC: detection and counting of small target *Cryptococcus* spp.

**DOI:** 10.3389/fcimb.2025.1701899

**Published:** 2025-12-16

**Authors:** Yanhui Chen, Yiwen Luo, Zan Yang, Longhua Hu, Qiaoshi Zhong, Tianle Sheng

**Affiliations:** 1 Jiangxi Province Key Laboratory of Immunology and Inflammation, Jiangxi Provincial Clinical Research Center for Laboratory Medicine, Department of Clinical Laboratory, The Second Affiliated Hospital, Jiangxi Medical College, Nanchang University, Nanchang, Jiangxi, China; 2 School of Advanced Manufacturing, Nanchang University, Nanchang, China

**Keywords:** *Cryptococcus* spp. detection, counting, attention-enhanced path aggregation network, hypergraph computation, diagnostic efficiency

## Abstract

**Introduction:**

*Cryptococcus* spp. infection can lead to cryptococcal meningitis (called CM) and pulmonary cryptococcosis, and how to diagnose *Cryptococcus* spp. infection accurately and timely is an urgent need in clinical practice. However, the existing methods such as cerebrospinal fluid (CSF) ink staining smear microscopy and CSF *Cryptococcus* spp. culture only rely on manual counting to determine the number of *Cryptococcus* spp., resulting in low efficiency. Thus, how to identify *Cryptococcus* spp. in cerebrospinal fluid and achieve automated counting of *Cryptococcus* spp. is of great significance for helping clinical experts accurately and timely diagnose *Cryptococcus* spp. infections to reduce the risk of deterioration.

**Method:**

We propose a small target *Cryptococcus* spp. detection and counting method called CsDETC, where three important components are integrated, such as data augmentation, hypergraph computation empowered semantic collecting and scattering module called HGC-SCS, and attention-enhanced path aggregation network called AEPAN. The *Cryptococcus* spp. dataset has been expanded through multiple data augmentation techniques such as random cropping, horizontal flipping, and rotation before training the model. Subsequently, the *Cryptococcus* spp. morphological features have been enriched by data augmentation based on perspective transformation and vertical flipping in the training process, thereby improving the generalization ability. Then different morphological features can be adaptively detected by learning high-order relationships between visual features when adding HGC-SCS into the neck network. Eventually, the convolution block attention module (CBAM) is integrated into path aggregation network to generate attention maps along the channel and spatial dimensions, transmitting more detailed information contained in the shallow layer to the deep layers to enhance the perception ability of small targets.

**Results:**

The experimental results on private dataset show that CsDETC outperforms other advanced object detection models with excellent performance such as YOLOv10 and YOLO11, etc. Typically, compared to the baseline, CsDETC shows significant improvements in mAP50 (93.6% vs. 91.3%), APs (51.0% vs. 49.5%), and MAE (1.865 vs. 2.370), while only a 0.7 millisecond increase in the inference time.

**Discussion:**

CsDETC is a promising tool that has performed well in preliminary validation. After validation with larger and more diverse datasets from different medical centers in the future, CsDETC can help doctors accurately and timely identify *Cryptococcus* spp. and achieve automated counting of *Cryptococcus* spp., providing reference for treatment plans and improving the diagnostic efficiency.

## 1 Introduction

*Cryptococcus* spp. infection can lead to cryptococcal meningitis (called CM) and pulmonary cryptococcosis (Zhang et al., 2023). Recently, the abuse or improper use of broad-spectrum antibiotics, hormones, and immunosuppressive drugs, as well as the significant increase in immunodeficiency diseases and organ transplant patients, have exacerbated the prevalence of such diseases (Jarvis et al., 2022). The atypical symptoms presented by these diseases have resulted in extremely high subjective misdiagnosis and treatment delay rates, with a mortality rate of up to 60% (Williamson et al., 2016). Therefore, how to diagnose *Cryptococcus* spp. infection accurately and timely is an urgent need in clinical practice.

The traditional detection methods for CM mainly include two categories: cerebrospinal fluid (CSF) ink staining smear microscopy and CSF *Cryptococcus* spp. culture (Hu et al., 2020). The ink staining method uses centrifugation to concentrate CSF for staining, which is simple and easy to implement and relatively fast but has low sensitivity (Xu et al., 2022). The CSF *Cryptococcus* spp. culture method is known as the “gold standard” for diagnosing CM, but the long growth cycle of *Cryptococcus* spp. leads to high time consumption, and the effectiveness of CSF culture methods will sharply decrease when infected with multiple pathogens (Temfack et al., 2021). Furthermore, some studies (Vidal et al., 2012; de Oliveira et al., 2022), have shown that CSF *Cryptococcus* spp. count is a valuable indicator for prognostic risk assessment of patients, but existing methods only rely on manual counting to determine the number of *Cryptococcus* spp., resulting in low efficiency. Therefore, how to identify *Cryptococcus* spp. accurately and timely in cerebrospinal fluid and achieve automated counting of *Cryptococcus* spp. is of great significance for effectively preventing the deterioration of *Cryptococcus* spp. infection and reducing the risk of prognosis.

In recent years, artificial intelligence detection technology has developed rapidly, and many AI technologies such as deep learning models have been successfully applied to pathogen morphology detection in clinical practice. These models can actively learn the core features inherent in medical images to achieve effective detection of specific targets in images. For example, in the field of pathology, Tan et al. (2024) used their RCM-YOLO model constructed based on the YOLOv5 architecture to detect small masses in mammography images, helping doctors screen breast images in batches, significantly improving the detection rate of small masses, and reducing misdiagnosis. This is of great significance for the early detection and treatment of diseases.

At present, few studies have focused on how to construct targeted deep learning models to detect *Cryptococcus* spp. Liu et al. (2022) directly using the YOLOv5 model to identify *Cryptococcus* spp. in images. After comparing YOLOv3, YOLOv5, YOLOv7, and CenterNet, Gu et al. (2023) chose the YOLOv5 as the benchmark model, where the C3 module is replaced with CSP in the backbone to ensure the depth of convolution, and the ResNet50 instead of non-maximum suppression is employed to analyze the calculation of bounding boxes in the output; both operations improve the accuracy of YOLOv5 and achieve effective detection of *Cryptococcus* spp. on proprietary datasets. The two studies focus on improving the model itself, while neglecting the design of corresponding architectures for features of *Cryptococcus* spp. images.

**Figure 1:**
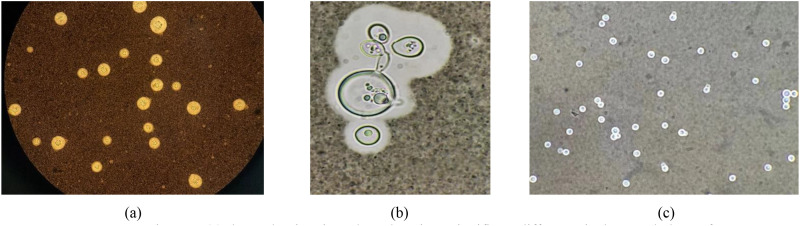
*Cryptococcus* spp. images. **(a)** shows the situation where there is no significant difference in the morphology of *Cryptococcus* spp. within the same image. **(b)** shows the significant differences in the morphology of *Cryptococcus* spp. within the same image. **(c)** shows the presence of many small targets in the *Cryptococcus* spp. image.

In fact, as shown in **Figure 1**, there are significant morphological differences among multiple *Cryptococcus* spp. in the same image, and there are many small target *Cryptococcus* spp. in the dataset. Furthermore, the number of *Cryptococcus* spp. in the image to some extent reflects the severity of the infection with *Cryptococcus* spp. However, the specific morphological characteristics of *Cryptococcus* spp. have not been considered in the existing studies to design suitable models. Moreover, they are unable to detect the number of *Cryptococcus* spp. in images directly, which cannot provide effective reference for the degree of *Cryptococcus* spp. infection in patient prognosis.

Considering that YOLOv8 is a state-of-the-art model in detecting small targets, this paper designs a *Cryptococcus* spp. detection and counting network called CsDETC based on the YOLOv8, in which the hypergraph computation empowered semantic collecting and scattering module (called HGC-SCS) and attention-enhanced path aggregation network (called AEPAN) are effectively integrated. To achieve adaptive detection for different morphological features of *Cryptococcus* spp., we introduce the HGC-SCS in the neck section to replace the original FPN structure. After fusing the feature maps generated in the five stages of backbone, HGC-SCS use hypergraph computation to model and learn complex high-order relationships between visual features, enhancing the ability to handle multi-scale problems and learn potential high-order correlations among visual features. High-order correlations refer to the complex and often non-linear relationships that exist among features at different scales, positions, and semantic levels, which are critical for understanding the deeper context and interactions within visual data (Feng et al., 2024). To further improve the detection performance of small targets, we introduce the convolution block attention module (CBAM) into the path aggregation network. CBAM generates attention maps along the channel and spatial dimensions in sequence, and then multiplies the attention maps with the input feature maps to perform adaptive feature refinement, allowing the model to focus more on small *Cryptococcus* spp. areas and enhance its perception ability of small targets. In addition, deep learning models typically require many images for training in order to fully exert their detection performance. However, the number of images of *Cryptococcus* spp. in clinical practice is limited, and these images cannot fully cover the diverse features of *Cryptococcus* spp. This may lead to poor performance of our designed model in training for *Cryptococcus* spp. images, thereby reducing the generalization ability. Thus, we designed a data augmentation module that can increase the diversity of *Cryptococcus* spp. images before training CsDETC. Specifically, offline augmentation is used before training to expand the dataset through data augmentation techniques such as random cropping, horizontal flipping, and rotation, as well as adding cell images of other microorganisms. During training, online augmentation is used to increase the diversity of *Cryptococcus* spp. by perspective transformation and vertical flipping, and the robustness of the model is enhanced by Mixup (Zhang et al., 2018).

Our contributions can be summarized as:

1. We use data augmentation to address the issue of insufficient sample size and enrich the morphological features of *Cryptococcus* spp. to improve the generalization ability of CsDETC.
2. We introduce the HGC-SCS in the neck section, which helps to transmit high-order messages in different semantic layers and positions of the backbone to enhance the visual backbone, thereby improving the ability to handle multi-scale problems and achieving adaptive detection for different morphological features of *Cryptococcus* spp.
3. We integrate the CBAM into the path aggregation network, which generates attention maps along the channel and spatial dimensions in sequence, giving more attention to the small *Cryptococcus* spp. areas and enhancing the perception ability of small targets.
4. We propose CsDETC to improve the object detection of *Cryptococcus* spp. and achieve the counting of *Cryptococcus* spp. Specifically, our CsDETC achieve significant improvements on the proprietary dataset, with mAP50 increasing by 2.3 percentage points and APs increasing by 1.5 percentage points compared to the YOLOv8s, while inference time only increased by 1.6 milliseconds. In terms of counting, the mean absolute error of CsDETC is 1.865, a decrease of 21.3% is achieved compared to YOLOv8s.

## 2 Materials and methods

### 2.1 YOLOv8 algorithm

Object detection is one of the core problems in the field of computer vision, whose task is to identify all objects of interest in an image and determine their categories and positions. At present, mainstream deep learning object detection algorithms are divided into two-stage detection algorithms (He et al., 2017; Ren et al., 2017; Cai and Vasconcelos, 2018) and one-stage detection algorithms (Liu et al., 2016; Redmon et al., 2016; Lin et al., 2020). The typical representative of two-stage detection algorithms is the Fast R-CNN (Girshick, 2015) series, which usually has high detection accuracy due to fine-grained processing of candidate regions. However, this type of method requires two-stage processing of candidate region generation and feature classification, resulting in a complex model structure, high computational complexity, and poor real-time performance. The typical representative of single-stage detection algorithms is the YOLO (Redmon and Farhadi, 2017; Redmon and Farhadi, 2018; Bochkovskiy et al., 2020; Li et al., 2022; Wang et al., 2023; Wang et al., 2024) (You Only Look Once) series of algorithms. The YOLO algorithm combines detection and classification tasks, with a simple model structure and strong real-time performance. The components of the YOLO series algorithm model include input, backbone, neck, and head. From YOLOv1 to YOLOv9, and their multiple improved versions, a balance between accuracy and speed has been achieved through improvements in backbone, multi-scale feature fusion, head, and anchor box mechanisms, making they widely used in various fields such as medicine, industry, and agriculture.

**Figure 2:**
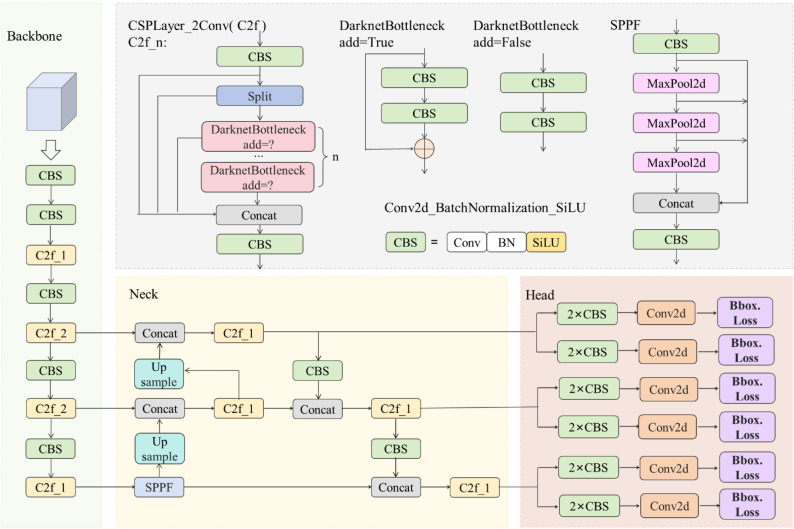
The network structure of YOLOv8s.

The YOLOv8 algorithm is built upon the foundation of the YOLOv5 model by incorporating the C2f module, decoupled-head design, anchor-free concept, Task-Aligned Assigner for sample matching, VFL Loss as the classification loss function, and DFL Loss + CIOU Loss as the regression loss function. This results in a YOLO-based model that achieves high precision and robustness, while maintaining fast detection speed. YOLOv8 is designed to be fast, accurate, and easy to use, making it an excellent choice for object detection, image segmentation, and image classification tasks. Like YOLOv5, YOLOv8 also offers models of different sizes at five scales (N/S/M/L/X) to meet the needs of different scenarios. Models of different sizes are acquired by adjusting the depth_multiple and width_multiple of parameters. Considering the cost of computing resources and running speed, YOLOv8s is used as the baseline model, with depth_multiple and width_multiple values of 0.33 and 0.50, respectively. The network structure of YOLOv8s is shown in **Figure 2**. The network structure of YOLOv8s is divided into four parts: input, backbone, neck, and head. In the input, YOLOv8 adopts the same Mosaic data augmentation, image size processing, and adaptive anchor box calculation as YOLOv5, and further introduces the operation of closing Mosaic in the last 10 epochs proposed in YOLOX (Ge et al., 2021), which can improve the generalization ability, optimize model performance, and reduce computational resource consumption. In the backbone, the C2f module is adopted as the basic constituent unit. The C2f module refers to the residual structure of the C3 module and the ELAN concept (Wang et al., 2023) to design a more complex gradient flow structure, and reduces computational complexity through cross-stage partial network. In the neck, the C2f module is integrated into the classic PAN-FAN structure to enhance the ability to extract and fuse features, thereby improving the detection capability for objects of different scales. In the head, the decoupled-head design is adopted to separates the classification head and bounding box regression head, which improves the training and inference efficiency of the network. In addition, YOLOv8 abandons the traditional anchor-based approach and adopts the anchor-free idea, further simplifying the model structure and reducing the hyperparameter settings of anchor boxes.

### 2.2 Small object detection

The detection of small objects in images is an essential key task in the field of computer vision, which has applications in various fields such as pathological cell recognition (Yu et al., 2021), plant detection (Zhao et al., 2021), intelligent transportation (Yang et al., 2023), remote sensing monitoring (Han et al., 2021), etc. Due to the less proportion of pixels, limited semantic information, susceptibility to complex scene interference, and easy aggregation and occlusion, small target detection has always been a major challenge in the field of object detection. There are usually two definitions of small object. Firstly, if the size of the object is less than 0.1 times the size of the original image, it is considered a relatively small target (Krishna and Jawahar, 2018). Secondly, in the MS-COCO (Microsoft common objects in context) dataset, if the size of the object is less than 32 × 32 pixels, it is considered an absolute small target (Lin et al., 2014).

The existing small object detection methods are all based on the improvement of mainstream object detection network models, which can be specifically divided into six categories. Ghiasi et al. (2021) designed a copy-paste data augmentation technique to address the shortage and uneven distribution of small object data. Compared with the state-of-the-art method on MS COCO, it achieved a relative improvement of 7.1% in object detection for small objects. Lin et al. (2017) proposed feature pyramid network (FPN), which combines low resolution strong semantic features with high-resolution weak semantic features using a top-down path and horizontal connections to enhance the information of feature maps. This is a multi-scale fusion method that addresses the weak representation ability of a single feature layer for small targets. After using FPN, Faster R-CNN achieved a 2.3% improvement in accuracy on the COCO dataset. Li et al. (2017) introduced the GAN method into small object detection tasks for the first time and proposed a perceptual generative adversarial network model (perceptual GAN). This model consists of two parts: a generator and a discriminator. By generating super-resolution representations of small targets, the difference in representation between small and large targets was narrowed, thus, the performance of small traffic sign detection got improved. Zhu et al. (2017) proposed a new fully convolutional network CoupleNet, which includes two different branches: Local Fully Convolutional Network (FCN) for extracting local information and Global FCN for extracting global information. In the end, local information, global information, and contextual information are fused to improve the detection performance of small targets. Focusing on small face detection, Zhang et al. (2017) proposed a new anchor densification strategy, which enables different kinds of anchors to have the same density on the image, thereby significantly improving the recall rate of small faces. Zhu et al. (2021) integrated convolution block attention module in their proposed TPH-YOLOv5 model to resist confusing information and quickly find the regions of interest, demonstrating good performance on the VisDrone2021 dataset. Given that the attention mechanism can resist confusing background information, quickly find regions of interest, and help the model obtain global spatial information of the feature map, we adopt the attention mechanism to improve the YOLOv8s benchmark model to enhance the detection accuracy of small *Cryptococcus* spp. in images.

### 2.3 Object detection for cell counting

Currently, there are two main cell counting methods proposed in the literature: one is density-based counting model, and the other is detection-based counting model. The density-based methods (Guo et al; Lempitsky and Zisserman, 2010; Cohen et al., 2017), represents the spatial distribution of targets by converting the input image into a continuous density map, thereby performing target counting. But the density-based methods use density maps instead of bounding boxes as labels, evading the difficult task of localization. In the second method, counting is typically done as a by-product of the predicted bounding boxes (Albuquerque et al., 2021; Drałus et al., 2021). It can also be done over the predicted segmentation masks (Morelli et al., 2021), but the workload brought by data annotation for segmentation will be enormous. The density-based methods are suitable for crowded scenes and cannot achieve the localization of *Cryptococcus* spp. in images. However, in the dataset used in this paper, only a very small part of the images contains dense objects. Therefore, the method of counting the predicted bounding boxes is adopted.

### 2.4 Overview of CsDETC

In order to improve the diagnostic efficiency for patients with *Cryptococcus* spp. infection, this paper designs a deep neural network model called CsDETC based on the YOLOv8s for identifying and automatically counting *Cryptococcus* spp. in cerebrospinal fluid staining images.

**Figure 3:**
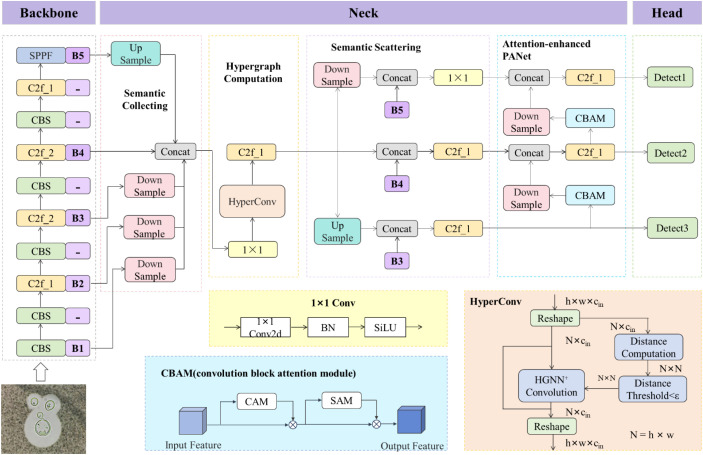
The structure of CsDETC.

The network architecture diagram of CsDETC is shown in **Figure 3**. Firstly, data augmentation methods such as perspective transformation and vertical flipping are used to enrich the morphological features of *Cryptococcus* spp. to improve the generalization ability, while Mosaic and Mixup are used to enhance the robustness. The backbone performs feature extraction on the input image and generates five stages of feature maps (B1, B2, B3, B4, B5) through down-sampling at 2, 4, 8, 16, and 32 times. These feature maps represent features at different semantic levels. Shallow feature maps have higher resolution but less semantic information, while deep feature maps have high-level semantic information but may lose some detail information due to multiple down-sampling processes. It is worth noting that the collaborative representation of low-level visual features and their correlations plays a crucial role in object detection tasks. To explore the high-order correlations behind low-level features for semantic analysis, the hypergraph computation empowered semantic collecting and scattering (HGC-SCS) module is used instead of the original feature pyramid network (FPN). The HGC-SCS module performs information fusion on the five stages of feature maps generated by the backbone, and then uses hypergraph computation to model and learn the complex high-order relationships among visual features. The generated high-order feature maps are fused with feature maps obtained through larger down-sampling multiples (B3, B4, B5) to generate semantic-enhanced visual representations, which provide a more comprehensive visual feature representation from the perspectives of semantics and high-order structure. With a small increase in computational complexity, the improvement of the HGC-SCS module elevates the proficiency of neck in extracting high-order features, enabling adaptive detection of *Cryptococcus* spp. with different morphological features and improving the detection accuracy of the model. The fused feature maps are fed into the attention-enhanced path aggregation network structure of the feature fusion module, where a large amount of invalid information is eliminated during the fusion process to further enhance the small target features. The neck network ultimately produces three feature maps of different scales, which are used to detect large, medium, and small targets, respectively. Finally, the feature maps of three sizes are fed into the head to complete category score prediction and regression of bounding box coordinate parameters, and the NMS algorithm is used to eliminate redundant prediction candidate boxes.

### 2.5 Data augmentation

Deep convolutional neural networks have performed exceptionally well in many computer vision tasks. However, these networks are heavily reliant on big data to avoid overfitting. Overfitting refers to the phenomenon where a network learns functions with very high variance to model the training data perfectly. Overfitting can lead to poor performance of the network on new data, i.e. poor generalization ability. Unfortunately, due to the privacy protection of patients, the need for medical expert labeling, and the enormous cost and manpower required for medical imaging, it is almost impossible to build a standard dataset that can contain a large number of *Cryptococcus* spp. images.

Data augmentation is a data space solution for solving the problem of limited data. It includes a set of techniques to enhance the size and quality of the dataset, which can significantly improve the richness of the samples, enable the model to learn better feature representations, and improve the detection accuracy of the model. Data augmentation can be divided into offline augmentation and online augmentation. Offline augmentation is completed before training the model. It first applies data augmentation techniques to the entire dataset, then saves all enhanced data, and finally loads the original data along with the enhanced data into the model for training. Online augmentation is performed during the model training process, and whenever an image is loaded into the model for training, data augmentation techniques are applied immediately. This means that the same image may be enhanced in different ways in different training epochs. Online augmentation provides higher flexibility and diversity, but it will increase the computational burden during training; offline augmentation can reduce the computational burden during training, but it requires more storage space, making it suitable for small datasets.

Both offline and online augmentation is used in this paper. Firstly, we use offline augmentation to expand the dataset through three data augmentation techniques: random cropping, horizontal flipping, and rotation. The online data augmentation techniques used in YOLOv8 include HSV color space adjustment, translation, scaling, horizontal flipping, and Mosaic. The perspective transformation and Mixup are added to the online data augmentation techniques used in this paper on the basis of YOLOv8, and the horizontal flipping is changed to vertical flipping.

**Figure 4:**
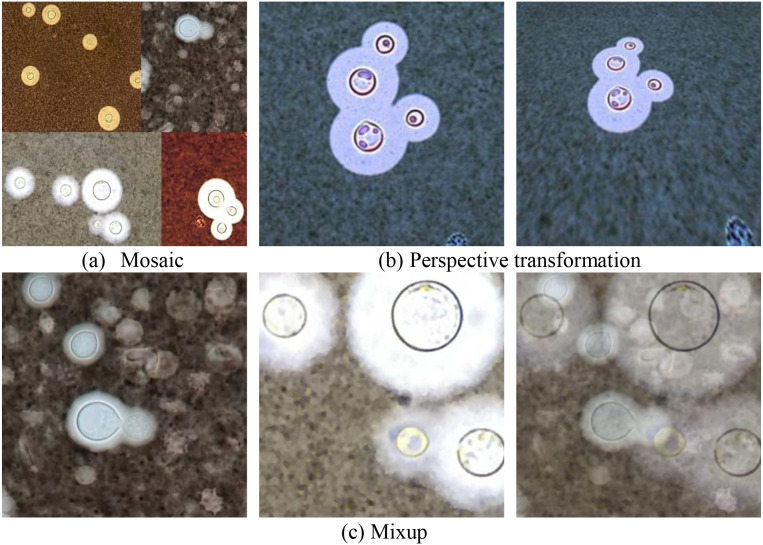
Data augmentation. **(a)** shows the outcome of the mosaic data augmentation method. **(b)** shows the outcome of the perspective transformation data augmentation method. **(c)** shows the outcome of the mixup data augmentation method.

The Mosaic method randomly selects four images from the training set, then performs random cropping and arrangement on these four images, and finally combines them together to form a new training sample, as shown in **Figure 4a**. Randomly selecting four images for combination, the resulting image contains more targets than the original image, thereby increasing the diversity of the data. Mixing four images with different semantic information allows the model to detect targets beyond the usual context, enhancing the robustness of the model.

Mixup is a data augmentation technique based on linear interpolation, which combines two different training samples to generate a new training sample, as shown in **Figure 4c**. Mixup improves the robustness of the model by introducing noise and perturbations to better adapt to unseen data.

Perspective transformation is mainly used to simulate the perspective effect of the human eyes or camera lens when viewing three-dimensional objects, thereby changing the perspective or shape of the object, as shown in **Figure 4b**. *Cryptococcus* spp. is mostly circular in shape, but there are also some individuals with significant differences in shape. By using two data augmentation techniques, perspective transformation and vertical flipping, we can obtain more *Cryptococcus* spp. with different shapes, increase the diversity of the dataset, and thus improve the generalization ability of the model.

### 2.6 Hypergraph computation empowered semantic collecting and scattering module

The *Cryptococcus* spp. in the dataset used in this paper exhibit significant differences in shape and size, posing an inevitable requirement on multi-scale feature fusion and representation method. Currently, classical models such as YOLOv5, YOLOv7, and YOLOv8 all adopt the traditional FPN+PAN structure in the neck network, promoting cross scale information fusion through top- down and bottom-up paths. However, the ability of FPN+PAN structure is mainly limited to fusing features between adjacent layers, and it cannot transmit information without loss when fusing features across layers, which hinders YOLO from achieving better information fusion. Moreover, YOLOv8 uses three feature maps with higher down-sampling multiples as input feature maps for the neck network, but these feature maps are prone to losing some information about small objects. In order to improve the detection performance of small objects, most methods (Zhu et al., 2021; Ji et al., 2023; Li et al., 2023) adopt the addition of an extra small object detection layer, but this approach leads to a sharp increase in computational complexity.

In response to the above issues, inspired by Hyper-YOLO (Feng et al., 2024), this paper adopts a hypergraph computation empowered semantic collecting and scattering module to replace the traditional FPN module. With a small increase in computational complexity, the detection accuracy of the model has been effectively improved. Firstly, to preserve more information about small objects, the HGC- SCS module concatenates five feature maps of different scales (*X*_1_, *X*_2_, *X*_3_, *X*_4_, *X*_5_) from the backbone by channel, and transposes the features extracted from the visual backbone into an abstract semantic space. To minimize computational complexity, a 1x1 convolution is used to compress the number of channels before performing hypergraph computation. Then, the hypergraph computation is utilized to enhance the feature maps extracted from the visual backbone. The hypergraph serves as a conduit for enabling high-order message propagation among features within the semantic space. Eventually, the generated high-order feature *X_hyper_* is fused with *X*_3_, *X*_4_, and *X*_5_, resulting in semantics-enhanced visual representations, $X_{3}^{\prime },X_{4}^{\prime }$, and $X_{5}^{\prime }$. These representations provide a more comprehensive visual feature representation from the perspectives of semantics and high-order correlation. The computational procedure of HGC-SCS module is shown in Equations 1–3:

(1) $$X_{mixed}=down\left(X_{1}\right)⊕down\left(X_{2}\right)⊕down\left(X_{3}\right)⊕X_{4}⊕up\left(X_{5}\right)$$

(2) $$X_{hyper}=C2f\left(HyperConv\left(Conv_{1\times 1}\left(X_{moxed}\right)\right)\right)$$

(3) $$\left\{{X^{\prime }}_{3},{X^{\prime }}_{4},{X^{\prime }}_{5}\right\}=\left\{X_{3}⊕up\left(X_{hyper}\right),X_{4}⊕X_{hyper},X_{5}⊕down\left(X_{hyper}\right)\right\}$$

where ⊕ denotes two feature map channel concatenation operations. down(·) denotes down sampling operation. up(·) denotes the bilinear interpolation operation. Conv1×1(·) denotes a convolution operation with the kernel size of 1.

The hypergraph computation includes two steps: hypergraph construction and hypergraph convolution.

**Figure 5:**
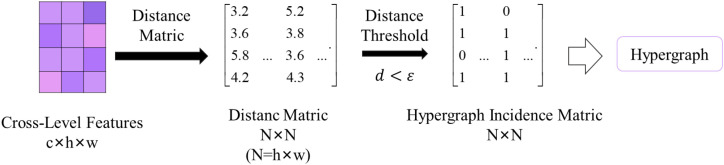
Distance-based hypergraph constructure.

1. Hypergraph Construction: A hypergraph *G*={*V*,*E*} is commonly defined by its vertex set *V* and hyperedge set *E*. HGC- SCS deconstructs the grid-based visual features to form the vertex set *V* of a hypergraph, where each pixel represents a vertex. To model the neighborhood relationships within the semantic space, a distance threshold ε is used to construct an ε-ball for each feature point, which serves as a hyperedge. The overall hyperedge set can be defined as *E* = {*ball* (*ν, ε*)|*ν* ∈ *V*}, where $ball(\nu ,\epsilon )=\{u|\|x_{u}-x_{u}{\|}_{d}<\;\epsilon ,u\in V\}$ denotes the neighbor vertex set of the vertex $\nu .\|x_{u}-x_{\nu }{\|}_{d}$ denotes the Euclidean distance between vertex *u* and vertex ν. ε is a trainable parameter. In computations, the hypergraph is usually represented by its incidence matric *H*. The process of hypergraph construction is shown in **Figure 5**.
2. Hypergraph Convolution: In order to facilitate the transmission of high-order messages on the hypergraph structure, spatial domain hypergraph convolution (Gao et al., 2023) and residual connection are used for high-order learning of vertex features. The formula for hypergraph convolution is shown in Equation 4:

(4) $$HyperConv\left(X,H\right)=X+D_{\nu }^{-1}HD_{e}^{-1}H^{T}X$$

where *X*represents the input feature map. *H* represents the incidence matrix of a hypergraph. *D _v_* and *D_e_* represent the diagonal degree matrices of vertices and hyperedges, respectively.

### 2.7 Attention-enhanced path aggregation network

Convolution block attention module (CBAM) is a simple but effective attention module. It is a lightweight universal module that can be integrated into any CNN architecture. In the *Cryptococcus* spp. dataset used in this paper, some images have complex background interference. The usage of CBAM can extract attention regions, helping CsDETC resist interference information and focus on useful target objects. More than that, CBAM can also help the model obtain global spatial information of the feature map, enriching the contextual semantic information in the feature map. Unlike the general attention mechanisms that rely solely on a single feature layer, the convolution block attention module is added to the network before each down-sampling of the feature map in the attention-enhanced path aggregation network (AEPAN), which passes more detailed information carried by shallow layers layer by layer to deep layers, thereby improving the small object detection accuracy of CsDETC.

**Figure 6:**
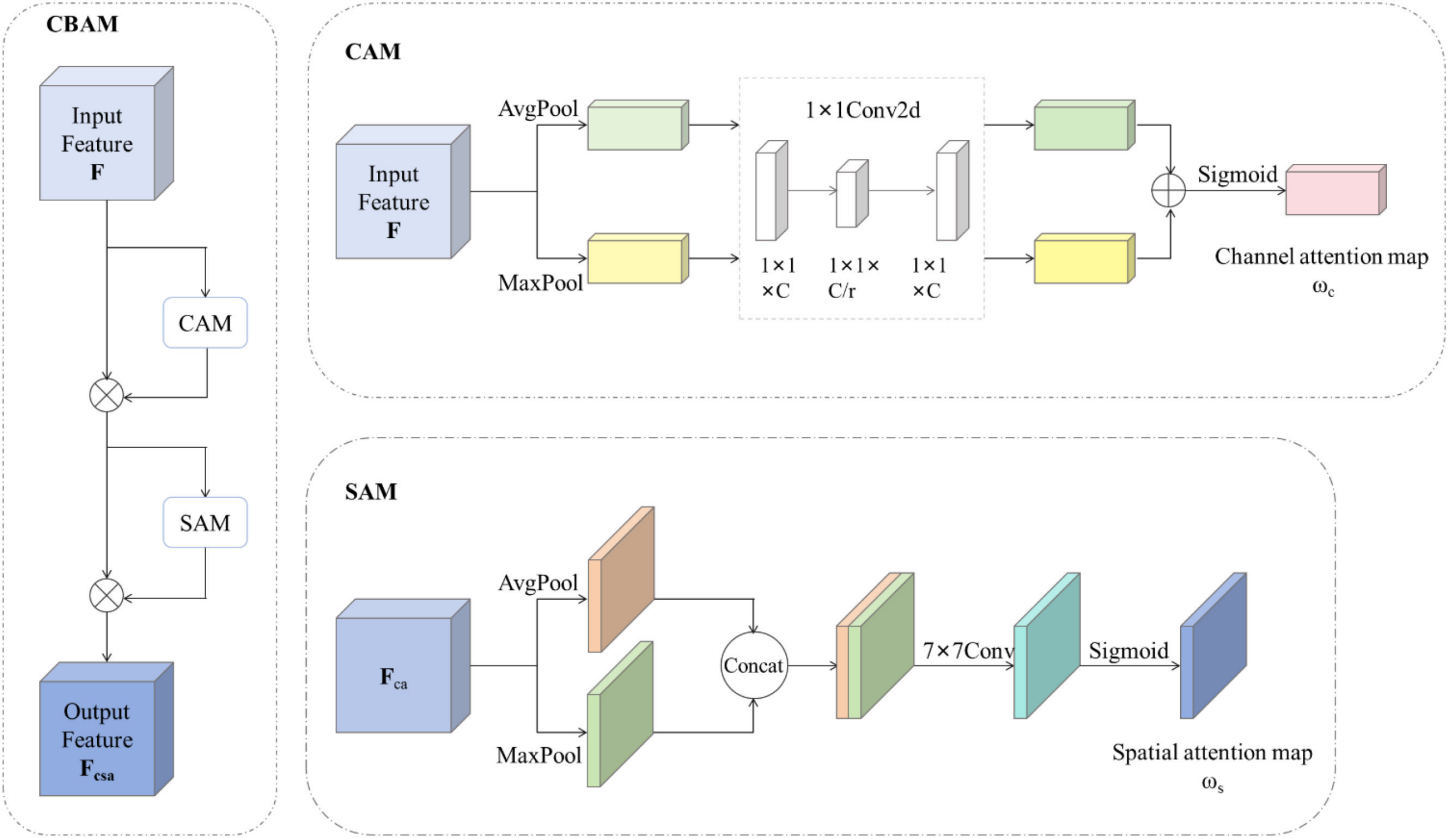
The internal structure of CBAM.

The CBAM consists of CAM (channel attention module) and SAM (spatial attention module), as shown in **Figure 6**. Given an input feature map *F* (C × H × W), in the CAM, *F* firstly compresses each channel feature layer through two parallel pooling layers such as MaxPool and AvgPool, resulting in two feature maps of size C × 1 × 1. Then the two feature maps are fed into the shared MLP module. In the shared MLP module, the number of channels of a feature map is first compressed to 1/r (Reduction) times its original value, then subjected to a ReLU activation function, and finally expanded to the original number of channels to obtain two output feature maps. This paper replaces the MLP operation in the shared MLP module with a more convenient convolution operation. The feature map obtained by adding the two output feature maps of the shared MLP module element by element is applied with a sigmoid activation function to obtain the channel attention weight matrix *ω_c_*. Finally, multiply this weight matrix with *F* to return to the size of C × H × W and obtain *F_ca_*. The calculation process of the channel attention mechanism is shown in Equations 5, 6:

(5) $$\omega_{c}=\sigma \left(Conv_{1\times 1}\left(AvgPool(F)\right)+Conv_{1\times 1}\left(MaxPool(F)\right)\right)$$

(6) $$F_{ca}=F⊗\omega_{c}$$

where σ denotes the sigmoid activation function. AvgPool(·) denotes the average pooling. MaxPool(·)denotes the maximum pooling. Conv_1×1_(·)denotes convolution operation with kernel size of 1. ⊗ denotes the operation of multiplying two feature maps by position.

In the SAM, the output result *F_ca_* of CAM is respectively applied the maximum and average pooling layers to obtain two feature maps of size 1 × H × W. The two feature maps are concatenated along the channel and the channels of the synthetic feature map is compressed into 1 by a convolution operation with kernel size of 7. Finally, a sigmoid activation function is used to obtain the spatial attention weight matrix *ω_s_*, and this weight matrix is multiplied by *F_ca_* back to the size of C×H ×W to obtain *F_csa_*. The calculation process of spatial attention mechanism is shown in Equations 7, 8:

(7) $$\omega_{s}\;=\sigma \left(Conv_{7\times 7}\left(AvgPool(F_{ca})\;⊕MaxPool(F_{ca})\right)\right)$$

(8) $$F_{csa}\;=F_{ca}⊗\omega_{s}$$

Where Conv_7×7_(·) denotes the 7×7 convolution operation. ⊕ denotes two feature map channel concatenation operations.

### 2.8 Dataset

**Figure 7:**
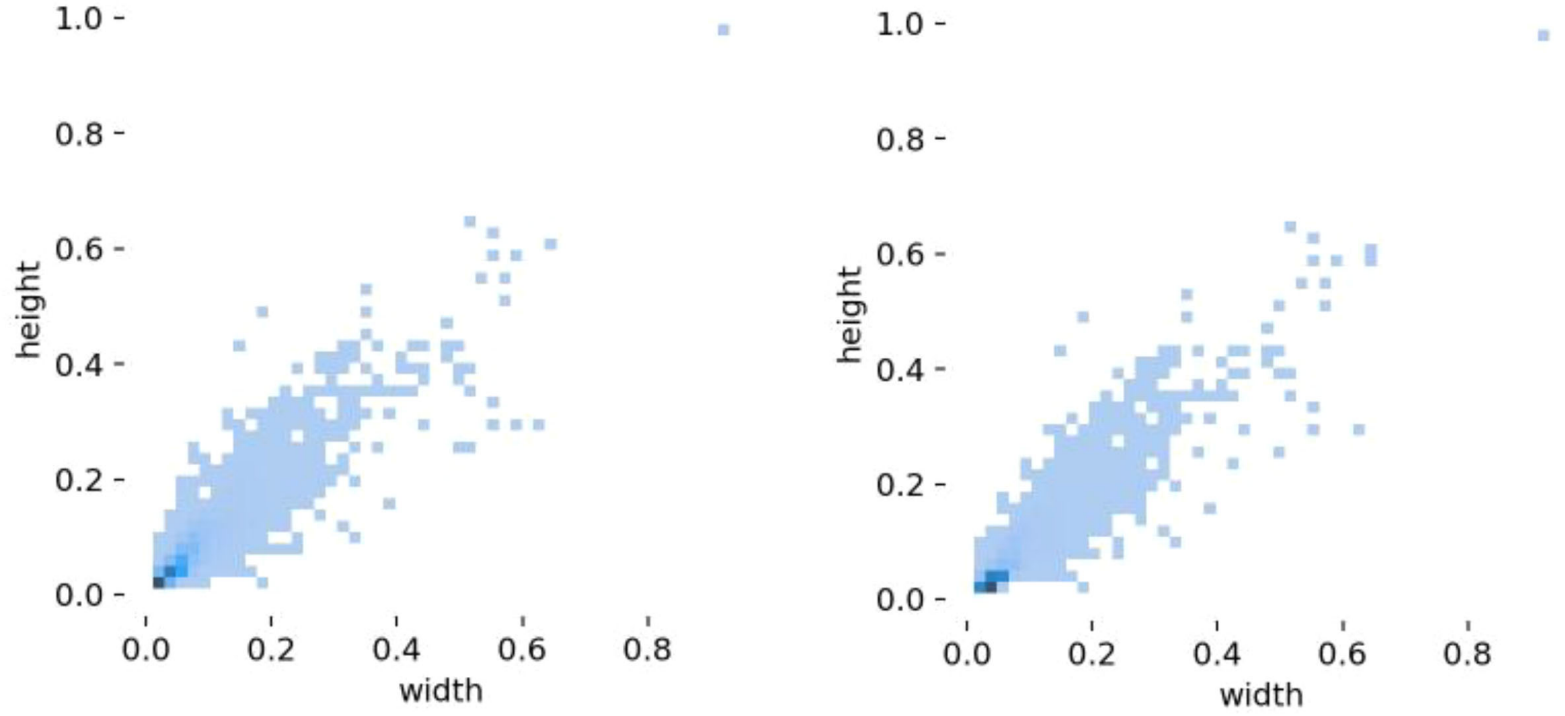
Training set length and width distribution of the initial dataset (left) and the complete dataset (right).

The dataset used in this article is provided by the Second Affiliated Hospital of Nanchang University. In the initial stage of this study, after adding digitally augmented images and some other microbial cell images similar to *Cryptococcus* spp., we constructed a dataset containing 1,000 images (hereinafter referred to as the initial dataset). To further enhance the rigor and persuasiveness of our research, we expanded the dataset with 2,025 images collected later. In the end, the complete dataset used in this study consisted of 3,025 images, and its sample size can more reliably support the training and evaluation of the model. From the length and width distribution of objects in the training set in **Figure 7**, it can be seen that our dataset contains a large number of small objects, which are relevant to the research problem of this paper. All regions of interest containing *Cryptococcus* spp. in the collected images are manually labeled by human pathologists using the labeling software Labelme. Finally, the annotated dataset is randomly divided into a training set and a testing set in an 8:2 ratios.

### 2.9 Model training and experimental setup

The model is programmed based on Python and Pytorch deep learning architecture, and the configuration of experimental environment is listed in **Table 1**. During the experiments, the training epoch value of the benchmark model is adjusted according to the dataset used in this paper. When using the original 300 epochs, we find that the loss function of the model has not fully converged after 300 epochs of training. It is determined through experiment that the best number of training epochs is 500. The settings of the training parameters and some hyperparameters for the model are shown in **Table 2**.

**Table 1:** Experimental environment configuration.

Experimental environment	Details
Operation system	Windows Server 2019 Standard 64 bit
Language	Python3.8
Deep learning architecture	Pytorch1.12.0
Acceleration environment	CUDA 11.3
CPU	Inter(R) Xeon(R) Gold 6348 3.5 GHz
GPU	Nvidia RTX A6000

**Table 2:** Model training parameters and hyperparameters.

Parameters	Details
Epochs	500
Batch size	8
Image size	640
Optimizer	AdamW
Initial learning rate	0.01
Momentum	0.937
Weight decay	0.0005

### 2.10 Evaluation metrics

The GFLOPs, Parameters, mAP50, mAP50-95, APS, APm, APL, and time are used as evaluation metrics for *Cryptococcus* spp. detection. The GFLOPs and Parameters are used to evaluate the complexity of the model. The fewer GFLOPs, the less hardware performance the model requires. The Parameters represents the total number of parameters used in the model, measured in millions (M).

The formulas for precision and recall are shown in Equations 9, 10:

(9) Precision=TP/(TP+FP)

(10) Recall=TP/(TP+FN)

where TP represents the number of *Cryptococcus* spp. samples that are correctly predicted as *Cryptococcus* spp. FP represents the number of samples that are not *Cryptococcus* spp. but are predicted to be *Cryptococcus* spp. FN represents the number of samples that are *Cryptococcus* spp. but are predicted to be non-*Cryptococcus* spp. The area under the Precision-Recall (PR) curve is defined as the average precision (AP), and mAP is obtained by averaging the AP values of all categories of targets. The higher the mAP value, the better the performance of the model (this article only needs to detect one category of target). Furthermore, mAP50 refers to the mAP calculated with an Intersection over Union (IoU) threshold of 0.50 between the ground truth and the predicted bounding box. In contrast, mAP50–95 represents the average mAP computed as the IoU threshold is progressively increased from 0.50 to 0.95 with a step size of 0.05. Additionally, APs, APm, and APL denote the AP for small, medium, and large targets, respectively, evaluated over an IoU range of 0.50 to 0.95. The small, medium, and large targets here use the COCO dataset standard. Targets with pixel areas less than 32 × 32 are classified as small targets, targets with pixel areas between 32 × 32 and 96 × 96 are classified as medium targets, and targets with pixel areas greater than 96 × 96 are classified as large targets. In this paper, we pay more attention to the APs because our dataset contains a large number of small targets. The calculation of AP and mAP is shown in Equations 11, 12:

(11) $$AP=\int_{0}^{1}P\left(r\right)dr$$

(12) $$mAP=(\sum_{i=1}^{n}AP_{i})/n$$

where P denotes the precision of the model. r denotes the recall of the model. n denotes the number of categories of targets. Time denotes the time taken to process an image during object detection, including the time spent on image preprocessing, inference, and non-maximum suppression algorithm. The less time it takes to process an image, the faster the detection speed of the model.

As for the counting of *Cryptococcus* spp., we used MAE (mean absolute error) as the evaluation metric. The decrease in MAE means that the estimated value of *Cryptococcus* spp. count in each field of view is closer to the true value. This may help doctors to more reliably monitor treatment response or assess disease severity. However, it should be pointed out that even if the error is reduced, there may still be clinical decision-making risks when the count is extremely low or high, which requires subsequent research to determine a clinically acceptable range of errors. The formula of MAE is shown in Equation 13:

(13) $$MAE=(\sum_{i=1}^{N}\left|P_{i}-G_{i}\right|)/N$$

where N represents the total number of images. P_i_ represents the number of targets predicted by the model in the i - th image. G_i_ denotes the true number of targets in the *i*-th image.

The model evaluation process mainly includes the following steps: Firstly, the model performs inference on the test set to generate prediction results, including bounding boxes, class probabilities, and confidence scores. Next, post-processing is applied to the predicted boxes using Non-Maximum Suppression (NMS) and confidence threshold filtering. Subsequently, the IoU between the predicted boxes and the ground truth boxes is calculated, and the correctness of the detections is determined based on a predefined IoU threshold (e.g., 0.50). Based on this, we compute Precision and Recall, plot the PR curve, and further calculate the Average Precision (AP) and the mean Average Precision (mAP) across all categories. To comprehensively evaluate the performance, we compute both mAP50 and mAP50-95. Additionally, we separately evaluate the performance for objects of different sizes (small objects APs, medium objects APm, and large objects APL). Finally, the MAE is calculated based on the target quantity of each image inferred by the model on the test set.

To validate the performance of the designed CsDETC or other key components in terms of statistical test, the Wilcoxon signed rank test (WT) suggested in Carrasco et al. (2020) and Derrac et al. (2011) is used to present the performance differences. The corresponding results are listed in the following **Tables 3**, **4** and **Tables 5**, **6** where P-value represents the probability of left-sided tests for the null hypothesis that the compared two datasets comes from a distribution with zero median, and H represents a logical value indicating the test decision when the significance level is equal to α. Specifically, H = 1 indicates a rejection of the null hypothesis, and H = 0 indicates a failure to reject the null hypothesis.

**Table 3:** Results of the attention module validity experiment.

Model	mAP50/%	mAP50-95/%	APs/%	APm/%	APL/%	WT			
						Tail Type	P-value	α	H
YOLOv8s (Model 1)	91.3	59.1	49.5	57.8	64.0	Left-sided	0.0312	0.05	1
YOLOv8s+DA+HGC-SCS (Model 2)	93.1	58.8	49.9	57.4	64.9	Left-sided	0.0312	0.05	1
YOLOv8s+DA+HGC-SCS+SE (Model 3)	92.9	59.5	49.5	58.7	64.5	Left-sided	0.0625	0.10	1
YOLOv8s+DA+HGC-SCS+ECA (Model 4)	93.3	59.8	50.4	**58.8**	65.6	Left-sided	0.2188	0.25	1
YOLOv8s+DA+HGC-SCS+CA (Model 5)	92.6	59.3	48.9	**58.8**	65.1	Left-sided	0.0625	0.10	1
YOLOv8s+DA+HGC-SCS+CBAM (CsDETC)	**93.6**	**60.1**	**51.0**	58.4	**65.8**				

The optimal values of the different models in terms of precision metrics are bolded.

**Table 4:** Results of the ablation experiment.

Number	Model	mAP50 /%	mAP50-95 /%	APs /%	APm /%	APL /%	GFLOPs	Time (ms)		WT		
									Tail Type	P-value	α	H
A	YOLOv8s	91.3	59.1	49.5	57.8	64.0	**28.4**	**4.8**	Left-sided	0.0312	0.05	1
B	A + DA	92.6	59.6	50.3	58.6	**66.0**	**28.4**	**4.8**	Left-sided	0.1562	0.20	1
C	A + HGC-SCS	91.9	59.9	48.6	**59.1**	64.8	31.0	5.0	Left-sided	0.0938	0.10	1
D	A + AEPAN	90.8	58.7	47.8	57.7	64.8	28.5	4.9	Left-sided	0.0312	0.05	1
E	B + HGC-SCS	93.1	58.8	49.9	57.4	64.9	31.0	5.3	Left-sided	0.0312	0.05	1
F	B + AEPAN	92.8	59.8	49.9	59.0	65.4	28.5	5.0	Left-sided	0.1562	0.20	1
G	E + AEPAN (CsDETC)	**93.6**	**60.1**	**51.0**	58.4	65.8	31.1	5.5				

The optimal values of the different models in terms of precision metrics are bolded.

**Table 5:** Detection results for the initial dataset.

Model	mAP50 /%	mAP50-95 /%	APs /%	APm /%	APL /%	GFLOPs	Time (ms)		WT		
								Tail Type	P-value	α	H
YOLOv5	90.2	56.9	45.3	55.6	62.6	8.0	3.2	Left-sided	0.0312	0.05	1
YOLOv7	90.3	54.1	45.5	54.1	62.0	**6.6**	**2.8**	Left-sided	0.0312	0.05	1
YOLOv8	91.3	59.1	49.5	57.8	64.0	14.3	4.8	Left-sided	0.0312	0.05	1
YOLOv9	90.4	58.8	46.9	**58.4**	64.0	19.8	6.5	Left-sided	0.0625	0.10	1
YOLOv10	90.2	58.9	47.6	57.9	62.5	12.4	4.7	Left-sided	0.0312	0.05	1
YOLO11	89.7	58.0	47.7	58.1	61.8	10.8	5.0	Left-sided	0.0312	0.05	1
TPH-YOLOv5	90.3	58.8	50.7	**58.4**	62.0	42.3	6.8	Left-sided	0.0625	0.10	1
Hyper-YOLO	91.6	58.8	46.9	58.1	64.5	19.6	6.3	Left-sided	0.0312	0.05	1
CsDETC	**93.6**	**60.1**	**51.0**	**58.4**	**65.8**	15.6	5.5				

Bold values highlight the best result for each metric across the models compared.

**Table 6:** Detection results for the complete dataset.

Model	mAP50 /%	mAP50-95 /%	APs /%	APm /%	APL /%	GFLOPs	Time (ms)		WT		
								Tail Type	P-value	α	H
YOLOv5	92.8	73.6	42.3	66.3	84.6	8.0	2.8	Left-sided	0.0312	0.05	1
YOLOv7	92.3	70.1	41.9	65.7	82.9	**6.6**	**2.7**	Left-sided	0.0312	0.05	1
YOLOv8	93.5	75.7	44.1	67.3	86.2	14.3	5.9	Left-sided	0.0625	0.10	1
YOLOv9	93.4	75.3	44.5	67.3	85.7	19.8	4.4	Left-sided	0.0312	0.05	1
YOLOv10	92.9	72.5	43.2	67.4	86.2	12.4	3.6	Left-sided	0.0625	0.10	1
YOLO11	93.1	75.0	43.4	67.6	86.0	10.8	6.6	Left-sided	0.0312	0.05	1
TPH-YOLOv5	93.1	71.2	45.1	67.5	83.4	42.3	7.6	Left-sided	0.0312	0.05	1
Hyper-YOLO	93.8	75.7	44.9	66.8	**86.7**	19.6	6.6	Left-sided	0.0938	0.10	1
CsDETC	**94.5**	**76.1**	**45.5**	**68.3**	86.2	15.6	4.8				

Bold values highlight the best result for each metric across the models compared.

## 3 Results

### 3.1 Module validity experiment

Different attention modules differ in their effects on the model, which requires further experimentation to ensure that the chosen attention module is beneficial for the model. Therefore, to verify the effectiveness of CBAM selected in the network in this paper, the CBAM module is utilized for comparison with the Squeeze-and-Excitation (SE) attention module (Hu et al), the Efficient Channel Attention (ECA) module (Wang et al., 2020), and the Coordinate Attention (CA) module (Hou et al., 2021). SE, ECA and CA are inserted into the same position of the model as CBAM. The results are shown in **Table 3**, where the optimal values of the different models in terms of precision metrics are bolded. Model 1 represent the YOLOv8s. Model 2 adds data augmentation and HGC-SCS modules on the basis of YOLOv8s. Models 3, 4, 5, and 6 have added SE, ECA, CA, and CBAM modules respectively on the basis of Model 2. In **Table 3**, five key evaluation metrics, i.e., mAP50, mAP50-95, APs, APm and APL of each model represent the overall performance on the initial *Cryptococcus* spp. dataset. Then these metrics of each model are considered as a performance dataset for this model, and the WT is used to obtain the performance differences between Model 6 and other models by comparing their respective performance datasets. It is obviously that the CsDETC shows a significant improvement over the Model 3 at the significance level α=0.10, over the Model 4 at α=0.25, over the Model 5 at α=0.10, and over both Model 1 and Model 2 at α=0.05. This means that the overall performance of the designed CsDETC performs better than all the variants.

From the **Table 3**, it can be seen that when CBAM is integrated into the path aggregation network, the mAP50, mAP50–95 and APs are increased by 0.5%, 1.3% and 1.1% respectively. This result fully validates the effectiveness of integrating the CBAM into the path aggregation network, as it significantly enhances the model’s ability to perceive small targets and suppress background interference.

### 3.2 Ablation experiment

To verify the effectiveness of additional data augmentation, HGC-SCS module, and AEPAN module, YOLOv8s is selected as the baseline model in this paper. Through ablation experiment under the same experimental condition, the impact of different modules and methods on object detection performance is evaluated when they are applied individually and in combination. The results of the ablation experiment are shown in **Table 4**. The DA represents data augmentation. Similar to section 3.1, the WT is conducted in **Tables 4** for obtaining the performance differences in terms of statistical test.

It is evident that the combination of data augmentation, HGC-SCS module, and AEPAN module shows significant improvement compared to the YOLOv8s at a significance level of α=0.05. This means that the overall performance of the designed CsDETC is significantly better than the original base model. Specifically, when the DA module or AEPAN module is integrated, the significance level is increased to 0.2, which means that both the DA and AEPAN provide significant positive improvements. Moreover, CsTETC performs significantly better than all the competitors, this directly demonstrate that all the modules can provide positive effects.

From the ablation experiment results in **Table 4**, it can be seen that the modules and methods proposed in this paper have improved the detection accuracy of the model and small targets to a certain extent. From the perspective of adding a module or method alone, experiment B improved all indicators except time after adding three data augmentation methods, namely perspective transformation, vertical flip and Mixup. The mAP50 increased by 1.3 percentage points, mAP50–95 increased by 0.5 percentage points, and APs increased by 0.8 percentage points. This indicates that enriching the diverse morphological features of *Cryptococcus* spp. and introducing some interferences appropriately are beneficial for improving the detection accuracy of the model. According to the experiment C, using HGC-SCS module instead of FPN module in the neck section led to a 0.6 and 0.8 percentage points improvement in mAP50 and mAP50-95, respectively, but a 0.9 percentage points decrease in APs, which indicates that learning high-order correlations among visual features through hypergraph computation can obtain better feature representations of the targets. However, due to the small pixel ratio and limited semantic information of small targets, modeling high-order relationships may introduce excessive noise or irrelevant information, resulting in a decrease in the ability of model to recognize small targets. The experiment D added the convolutional block attention module to the path aggregation network of YOLOv8s, which improved the detection accuracy of large targets by 0.8 percentage points, while all other indicators slightly decreased. This may be due to the fact that the dataset contains a large number of small targets, but the network gradually loses some of the detailed information of these small targets during feature extraction, resulting in a decrease in the detection accuracy. From the perspective of combining different modules and methods, experiments E, F, and G show that although combining data augmentation with the HGC-SCS module reduced mAP50–95 and APm by 0.3 and 0.4 percentage points, respectively, all accuracy indicators of the model are significantly improved when the three modules are combined. The mAP50 raised by 2.3 percentage points, mAP50–95 raised by 1 percentage point, and APs, APm, and APL raised by 1.5, 0.6, and 1.8 percentage points, respectively. The HGC-SCS module not only learns high-order correlations among visual features, but also concatenates five feature maps of different scales come from the backbone along channel, which preserves more localization information about small targets. Then, the AEPAN module enhances the perception ability of small targets by introducing the convolutional block attention module to extract attention regions. Therefore, the CsDETC model, which integrates three aspects of improvement, can effectively complete the task of detecting small targets in cerebrospinal fluid *Cryptococcus* spp. images.

### 3.3 Comparison experiment

To further validate the superiority of the CsDETC model, in addition to conducting comparison experiments with YOLO series algorithms such as YOLOv5, YOLOv7, YOLOv9, YOLOv10, and YOLO11, it is also compared with the award-winning model TPH-YOLOv5 from the VisDrone dataset competition and Hyper-YOLO, which integrates hypergraph computation into object detection algorithms. The results of comparison experiments are shown in **Table 5**. To demonstrate that the performance we report is not a random lucky run, our model undergoes 10 independent trainings on all datasets and the averages are taken as the final results. Similar to section 3.1, the WT is conducted in **Tables 5**, **6** for obtaining the performance differences. It is obviously that the designed CsDETC shows a significant improvement at the significance level of α≤0.1 compared to all other comparative models. This means that the overall performance of the designed CsDETC is significantly better than the other models.

As can be seen from **Table 5**, the CsDETC model achieved the highest detection accuracy compared to other algorithm models while maintaining high detection speed requirements. The mAP50 and mAP50–95 reached 93.6% and 60.1%, respectively, while APs, APm, and APL reached 51.0%, 58.4%, and 65.8%, respectively. Compared with the classic YOLOv5 and YOLOv7, although the speed of CsDETC has decreased, its various accuracy metrics have obvious advantages. Compared to the latest models YOLOv9, YOLOv10, and YOLO11 launched by the YOLO series in 2024, the CsDETC proposed in this paper has significantly better detection accuracy than these models and surpasses YOLOv9 in detection speed. Compared with the TPH-YOLOv5 model, although its performance in APs and APm metrics is comparable to the CsDETC proposed in this paper, CsDETC exhibits better performance in mAP50 and mAP50–95 metrics. Furthermore, TPH-YOLOv5 has significantly higher computational complexity than CsDETC, resulting in relatively slower detection speed. Compared to the Hyper-YOLO model, the CsDETC model exhibits superior performance in both detection accuracy and speed.

To further strengthen the conclusions of this study, we expanded the dataset to 3,025 images and conducted all comparison experiments again on this complete dataset. This move aims to evaluate the performance of the models on larger data scales and examine the generalization ability and robustness of our proposed CsDETC. The newly added comparative experimental results are shown in **Table 6**. As shown in **Table 6**, our proposed CsDETC consistently outperforms all comparison models on a larger dataset. Compared with the results on the initial dataset, the performance of all models has significantly improved, which confirms the importance of data size for model performance. More importantly, CsDETC has a more significant and stable performance advantage at this data scale, which strongly demonstrates its excellent scalability and robustness.

### 3.4 Results of counting

This section compared the counting performance of CsDETC model and other YOLO models on the testing sets, and the results are shown in **Table 7** and **Table 8**. It is worth noting that as long as there is a partial of nucleus, it is considered as a *Cryptococcus* spp. The CsDETC model proposed in this paper has a MAE of 1.865, representing a 21.3% reduction compared to the 2.370 MAE of YOLOv8. More than that, compared to other YOLO models, CsDETC achieves the smallest MAE. It suggests that the CsDETC model is superior to the YOLOv8 model and other YOLO models in terms of counting.

**Table 7:** Counting results for the original dataset.

Model	YOLOv5	YOLOv7	YOLOv8	YOLOv9	YOLOv10	YOLO11	CsDETC
MAE	1.980	2.905	2.370	2.655	2.230	3.400	**1.865**

Bold values highlight the best result for each metric across the models compared.

**Table 8:** Counting results for the complete dataset.

Model	YOLOv5	YOLOv7	YOLOv8	YOLOv9	YOLOv10	YOLO11	CsDETC
MAE	3.169	3.283	3.215	3.210	3.319	3.321	**3.152**

Bold values highlight the best result for each metric across the models compared.

### 3.5 Visualizations of feature maps

**Figure 8:**
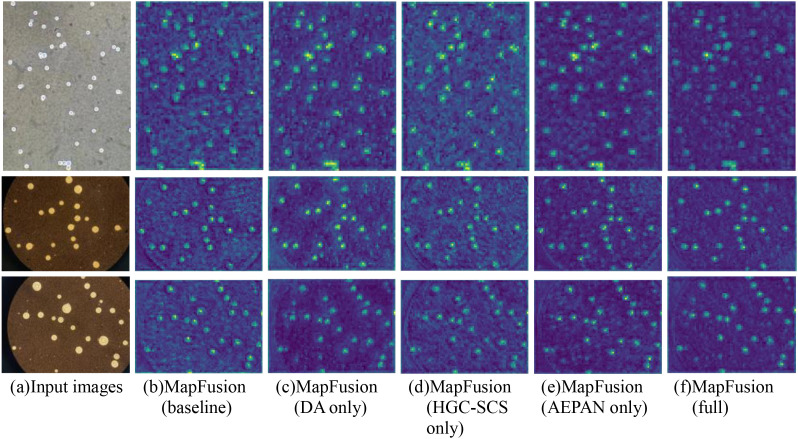
Comparison results of feature maps visualization. **(a)** shows the input images. **(b)** shows the response intensity of feature maps on model YOLOv8s. **(c)** shows the response intensity of feature maps on model YOLOv8s+DA. **(d)** shows the response intensity of feature maps on model YOLOv8s+HGC-SCS. **(e)** shows the response intensity of feature maps on model YOLOv8s+AEPAN. **(f)** shows the response intensity of feature maps on model CsDETC.

To further validate the effectiveness of the proposed improvement modules, a visual analysis of the feature maps is conducted. By comparing the feature maps of the baseline model (YOLOv8s) and the improved model (YOLOv8s+DA, YOLOv8s+HGC- SCS, YOLOv8s+AEPAN, CsDETC), we can intuitively observe the impact of different improvement modules on the feature extraction ability. The feature fusion layer in Neck is chosen for visualization because the feature map in this layer contains both high-level semantic information and more detailed information from shallow layers, which can reflect the overall understanding ability of the model towards the target. Specifically, channel activation maps are used to display the response intensity of feature maps, where brighter colors indicate higher activation values and darker colors indicate lower activation values. We conduct inference on three test images and extract feature maps from different models for comparison. The comparison results are shown in **Figure 8**.

From **Figure 8**, it can be observed that the feature map of the baseline model exhibits a relatively scattered response to the target, with some noise activation in the background regions, indicating limited localization capability of the model. When data augmentation is applied, the response of the feature maps to the target becomes more concentrated, and the background noise is significantly reduced, demonstrating that data augmentation effectively enhances the model’s robustness to *Cryptococcus* spp. features. After incorporating the HGC-SCS module, the activation intensity in the target regions of the feature map is noticeably enhanced, indicating that the HGC-SCS module can better extract the salient features of *Cryptococcus* spp. However, it is observed that the addition of the HGC-SCS module introduces some noise. With the use of the AEPAN module, the response of the feature map at the edges of the target becomes clearer, and the background noise is significantly reduced, suggesting that the AEPAN module can effectively focus on attention regions while ignoring background interference. When all improvement methods are combined, the feature map shows the most concentrated response to the target and the least background noise, indicating that the integration of data augmentation, the HGC-SCS module, and the AEPAN module significantly enhances the model’s feature extraction capability. The above visualization results are consistent with the performance improvement of ablation experiment, which further verifies the effectiveness of the proposed improvement modules.

### 3.6 Model convergence and generalization analysis

**Figure 9:**
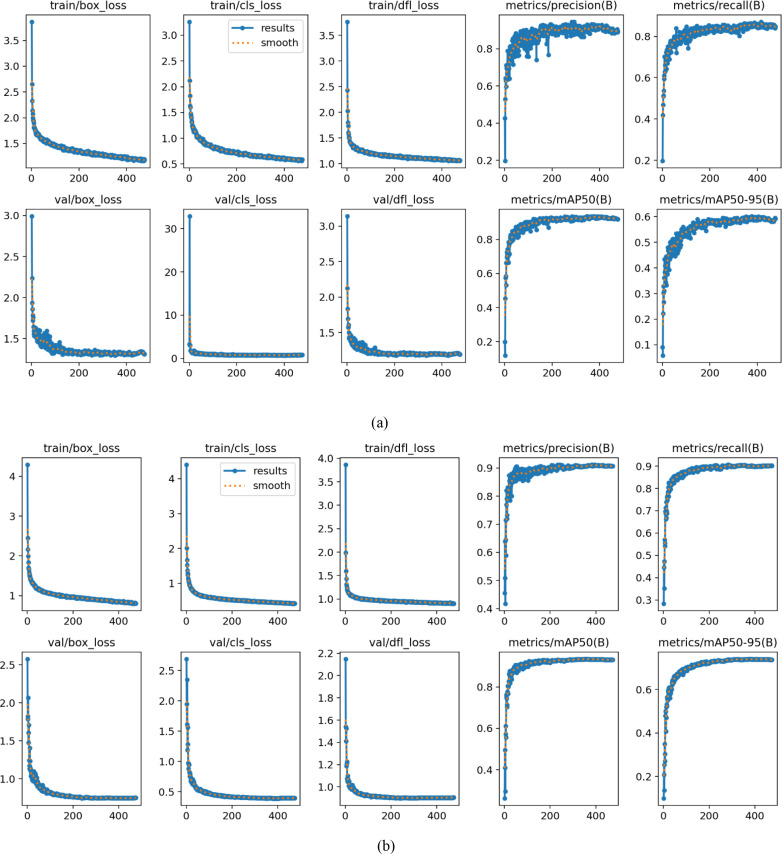
Learning curves. **(a)** Learning curves for the initial dataset. **(b)** Learning curves for the complete dataset.

To validate the convergence of CsDETC during the training process, we plot the learning curves for both the training and testing sets, as shown in **Figure 9**. This figure illustrates the progression of loss, mAP50, and mAP50–95 metrics with the epochs for both datasets. From **Figure 9**, it can be seen that the loss curves on both the training and testing sets smoothly decrease and tend to stabilize. Moreover, the performance of the testing set steadily improves with the training period and ultimately stabilizes at a high level. Based on the above observations, we can conclude that although the model is trained on small datasets, it exhibits good generalization ability and the reported high performance is reliable.

## 4 Discussion

In this study, we propose several improvements to the YOLOv8 architecture, including the data augmentation techniques, the HGC-SCS module and the AEPAN module, to enhance its performance in detecting *Cryptococcus* spp. Our experimental results demonstrate that the proposed improvements significantly boost the performance of the model, achieving a 2.3% increase in mAP50, a 1% increase in mAP50–95 and a 1.5% increase in APs compared to the baseline YOLOv8 model. These results validate the effectiveness of our approach in addressing key challenges such as small object detection and false positives.

**Figure 10:**
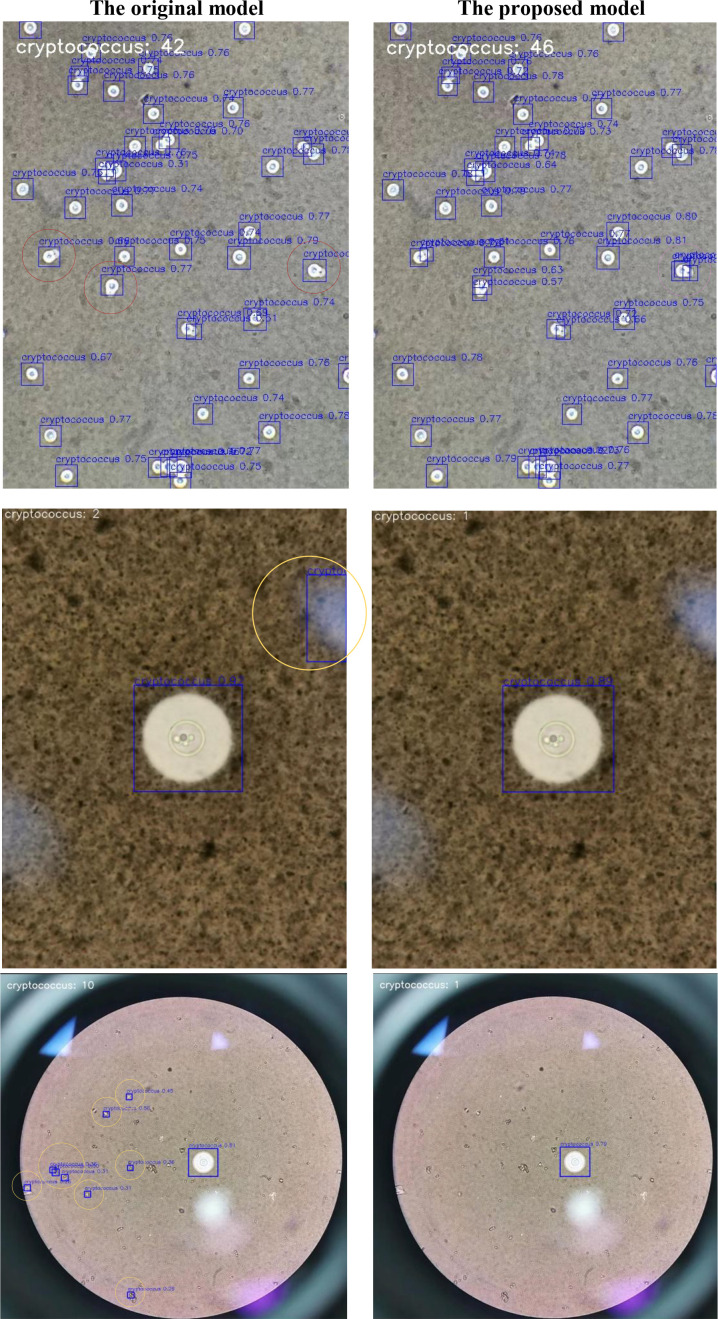
Comparison of detection results between the original model and our improved model. The missed detection areas of the models are marked with red circles, the false positive areas are marked with yellow circles, and the duplicate detection areas are marked with pink circles.

The detection results further illustrate the advantages of our proposed improvements. As shown in **Figure 10**, the original model fails to detect several small targets (marked with red circles), whereas our proposed model successfully identifies these targets. Moreover, the original model generates multiple false positives in background regions (marked with golden circles), while our proposed model significantly reduces these errors. However, we also find that the improved model has the problem of duplicate detection in some cases (marked with pink circles), which may be caused by overlapping target boundaries.

Compared to the baseline YOLOv8 model and other state-of-the-art such as YOLOv9, TPH-YOLOv5, Hyper-YOLO and so on, our proposed model achieves superior performance across all accuracy evaluation metrics, especially true for the smaller objects which have been the focus of this study.

Note that, while this study focuses on improving the YOLOv8 model according to the *Cryptococcus* spp. features, the proposed model in this paper may be applicable to other medical imaging tasks, such as the detection of small breast masses, the detection and counting of the blood cells, and so on. Firstly, the HGC-SCS module proposed in this paper, by integrating feature map information from different stages of the backbone and leveraging hypergraph computation mechanism, can effectively capture the features of multi-scale targets in medical images. This is particularly important for tasks such as detecting small breast masses, as these targets are typically small in size and exhibit significant morphological variations. Secondly, the AEPAN module, by introducing an attention mechanism, significantly enhances the model’s ability to perceive target edges and details, which is of great value for accurately segmenting and localizing small targets in medical images, such as breast masses and pulmonary nodules. Additionally, the counting function proposed in this study can effectively improve the efficiency of tasks such as blood cell counting. Therefore, the model proposed in this paper are not only suitable for the detection and counting of *Cryptococcus* spp. but can also be extended to other medical image analysis tasks, providing a universal solution for research in related fields.

Despite the promising results, our approach has certain limitations. For example, the addition of the HGC-SCS and AEPAN modules increases the computational cost, which may limit its applicability in real-time scenarios with strict latency requirements. Additionally, while the model we propose is an improvement on the convolutional neural network architecture, its generalization ability to other architectures such as Transformer-based models remains to be validated. Future work could focus on optimizing the model’s efficiency and exploring its adaptability to diverse architectures. For example, investigate techniques to reduce the computational overhead of the proposed modules without sacrificing performance and explore the potential of integrating our proposed modules with other advanced detection frameworks to achieve even greater performance improvements.

## 5 Conclusion and limitation

Aiming at the problem of low diagnostic efficiency of *Cryptococcus* spp. infection, this paper proposes a small target *Cryptococcus* spp. detection and counting method CsDETC. The method proposed in this paper mainly includes three aspects of improvement: data augmentation, improvement of the neck, and introduction of attention mechanism. For the first one, the improvement of data augmentation refers to the use of perspective transformation and vertical flipping to enrich the morphological diversity of *Cryptococcus* spp. for improving the generalization ability of the model, as well as the use of Mixup to enhance the robustness of the model. In the ablation experiment in Section 4.4, it can be seen that the improved data augmentation method effectively improves the performance of the model on all accuracy metrics without increasing the complexity of the model. For the second one, the improvement of the neck means replacing the traditional FPN module with the HGC-SCS module, which learns high-order correlations among visual features through hypergraph computation to obtain better feature representations, while performing channel wise concatenation operation on five feature maps of different sizes from the backbone to preserve more detailed information about small targets and facilitate information interaction across layer. Experiment C in Section 4.4 shows that the HGC-SCS module learns better feature representations and improves the detection accuracy of the model. Finally, the introduction of attention mechanism refers to adding a convolution block attention module before each down-sampling module in the path aggregation network to extract attention regions and enhance the perception ability of small targets. From the experimental results, it can be seen that introducing attention mechanism significantly improves the detection accuracy of the model with minimal increase in computational complexity.

In summary, our model achieves the best detection accuracy compared to classic, latest YOLO models and other YOLO improved models. From the results of comparison experiment, it can be seen that the model proposed in this paper can achieve 93.6% mAP50, 60.1% mAP50–95 and 51.0% APs.

CsDETC is a technically promising deep learning approach to the task for detection and counting of *Cryptococcus* spp., which has performed well in preliminary validation. However, we also find that there are some limitations in this model. For example, the problem of duplicate detection still exists in the case of target aggregation and occlusion, although it has improved compared to the original model. Besides, the increased computational cost of the proposed modules may limit real-time applications. Moreover, the relatively small size of the dataset-just 3,025 images from one institution may affect the performance and robust of the model. Furthermore, in practical clinical scenarios, *Cryptococcus* spp. images may have other complex characteristics such as noise, blur, and different staining conditions; Thus, how to design de-noising modules and how to increase image clarity to design targeted detection models are also important research directions in the future. Finally, the potential integration of the proposed modules with other advanced architectures such as Transformer-based models, represents an unexplored yet promising direction for future research. Future work could focus on the above aspects.

## Data Availability

The original contributions presented in the study are included in the article/supplementary material. Further inquiries can be directed to the corresponding author/s.
